# Reduced Cervical Muscle Fat Infiltrate Is Associated with Self-Reported Recovery from Chronic Idiopathic Neck Pain Over Six Months: A Magnetic Resonance Imaging Longitudinal Cohort Study

**DOI:** 10.3390/jcm13154485

**Published:** 2024-07-31

**Authors:** Suzanne J. Snodgrass, Kenneth A. Weber, Evert O. Wesselink, Peter Stanwell, James M. Elliott

**Affiliations:** 1 Discipline of Physiotherapy, The University of Newcastle, Callaghan 2308, Australia; 2 Centre for Active Living and Learning, Hunter Medical Research Institute, New Lambton Heights 2305, Australia; 3 Department of Anesthesiology, Perioperative and Pain Medicine, Stanford University School of Medicine, Stanford, CA 94305, USA; kenweber@stanford.edu; 4 Vrije Universiteit Amsterdam, 1081 HV Amsterdam, The Netherlands; e.o.wesselink@vu.nl; 5 Discipline of Medical Radiation Science (Diagnostic Radiography), The University of Newcastle, Callaghan 2308, Australia; peter.stanwell@newcastle.edu.au; 6 The Kolling Institute, Northern Sydney (Arabanoo) Precinct, St Leonards 2065, Australia; james.elliott@sydney.edu.au; 7 Sydney School of Health Sciences, The University of Sydney, Camperdown 2050, Australia

**Keywords:** muscles, neck muscles, muscle, skeletal, neck pain, chronic pain, musculoskeletal pain, intramuscular adipose tissue (IMAT), fat

## Abstract

**Background:** It is unclear why neck pain persists or resolves, making assessment and management decisions challenging. Muscle composition, particularly muscle fat infiltrate (MFI), is related to neck pain, but it is unknown whether MFI changes with recovery following targeted interventions. **Methods:** We compared muscle composition quantified from fat-water magnetic resonance images from the C3 to T1 vertebrae in individuals with and without chronic idiopathic neck pain at two times 6 months apart. Those with neck pain received six weeks of intervention (physiotherapy or chiropractic) after their baseline MRI; at 6 months, they were classified as recovered (≥3 on the 11-point Global Rating of Change scale) or not recovered. **Results:** At 6 months, both asymptomatic and recovered individuals had decreased MFI compared to baseline (asymptomatic estimated marginal mean difference −1.6% 95%; CI −1.9, −1.4; recovered −1.6; −1.8, −1.4; *p* < 0.001) whereas those classified as not recovered had increased MFI compared to baseline (0.4; 0.1, 0.7; *p* = 0.014), independent of age, sex and body mass index. **Conclusions:** It appears MFI decreases with recovery from neck pain but increases when neck pain persists. The relationship between cervical MFI and neck pain suggests MFI may inform diagnosis, theragnosis and prognosis in individuals with neck pain. Future development of a clinical test for MFI may assist in identifying patients who will benefit from targeted muscle intervention, improving outcomes.

## 1. Introduction

Neck pain is a common problem with a high burden for individuals, healthcare systems and society [1]. Neck pain ranks in the top 11th of 369 health conditions in terms of years lived with disability [2], and when combined with low back pain, it is the leading cause of years lived with disability worldwide [3]. Globally, the years lived with neck pain-related disability has increased by 75% over the last 30 years [4]. The high prevalence rate [5] of neck pain is also increasing [1,6] and is expected to continue to rise, largely due to population growth, ageing [4] and potentially due to increases in overweight and obesity [1], Interventions that provide long term relief for neck pain remain elusive. Symptoms commonly recur [7], suggesting that current intervention approaches may not address the source of symptoms. One of the most common types of neck pain, termed non-specific or idiopathic neck pain, is characterised as having no identifiable cause of pain, as radiological investigations typically do not correlate with patient symptoms [8]. Thus, there is a need for investigations to understand the underlying contributors to neck pain so that new innovations, diagnostics and management strategies can be designed to target the underlying problem more effectively.

Muscle health is one possible biological mechanism that may affect the onset, persistence, and recovery from neck pain. The cervical muscles are commonly implicated as a source of dysfunction in a wide range of disorders, with anatomical, mechanical or functional associations to the jaw [9], eyes [10] and thoracic spine [11]. Muscle health is typically described by muscle size (cross-sectional area [CSA] or volume measured by imaging) and muscle composition. [12]. Muscle fat infiltrate (MFI) is the estimated fat within the muscle, quantified using signal intensities from the water and fat images produced using multi-echo MRI acquisitions (e.g., Dixon technique) [13,14]. Muscle volume and MFI can be used to calculate ‘relative muscle volume’ representing lean muscle mass: the amount of tissue within the muscle boundaries that can be attributed to muscle mass (without fat or fascial tissue). A better understanding of muscle composition may contribute to more targeted therapies to resolve neck pain.

Altered muscle composition has been associated with having neck pain [15]. Greater volume and MFI, with less relative volume, are observed in individuals with neck pain compared to asymptomatic populations [16,17]. Greater cervical MFI is consistently demonstrated in individuals with whiplash-associated disorder (WAD) [18,19,20,21,22] and appears to be localised to the cervical spine and not a generalised increase in muscle fat throughout the body [23,24]. The majority of studies of muscle composition in neck pain investigate individuals with WAD [25] or, when including idiopathic neck pain, limit their populations to females [16,17]. Two studies report muscle composition specifically in individuals with idiopathic neck pain [26,27], though only one of these includes MFI, reporting greater MFI in the multifidus of individuals with idiopathic neck pain compared to asymptomatic controls [27]. Thus, further studies of muscle composition in idiopathic neck pain are needed.

Relationships between chronic pain and MFI appear to lie along a continuum. For example, greater MFI in the cervical multifidus was observed in those with greater disability (>30% neck disability index [NDI]) compared to asymptomatic controls, but those with mild disability (<30% NDI) had similar MFI to controls [24]. This continuum suggests that muscle composition, particularly MFI, might be related to changes in pain or predict pain recovery. However, very few studies on any type of neck pain investigate whether muscle composition changes over time or in relation to changes in pain. One study showed that individuals with WAD had greater CSA in multifidus than asymptomatic controls at baseline and 10 years follow-up and greater CSA in semispinalis cervicis and semispinalis capitis compared to controls at follow-up [28]. However, both groups changed similarly over time (small increase), with the authors reporting this was likely due to ageing [28]. One single-group cohort study of 5 females with chronic WAD showed that MFI in the multifidus might decrease following 10 weeks of supervised exercise [29]. These preliminary findings suggest muscle composition may be one biomarker of neck pain that might be addressed via interventions that lead to decreased symptoms. For example, motor control training has been shown to improve neck pain and disability [30], presumably through an improvement in motor performance that has led to recovery. The lack of longitudinal studies of muscle composition and the diagnostic potential of muscle composition indicate a clear need for investigations to understand variations in muscle composition over time and whether these are related to recovery. This will support the development of diagnostic tests and interventions that include and address muscle composition.

Therefore, the aim of this study was to determine whether muscle composition (MFI, volume and relative volume) changes over six months in individuals with and without chronic idiopathic neck pain and whether changes are associated with recovery from neck pain. We hypothesised that muscle composition would improve (less MFI, greater lean muscle mass represented by relative volume) in individuals with neck pain who recovered over six months.

## 2. Materials and Methods

This longitudinal cohort study measured muscle composition in individuals with chronic idiopathic neck pain ≥ 3 months and age and sex-matched asymptomatic controls at baseline and 6 months. The study population was drawn from two studies investigating the biological effects of physiotherapy (*n* = 42) and chiropractic (*n* = 21) interventions in individuals with idiopathic neck pain. Each study included age and sex-matched asymptomatic controls to investigate normal variability over time (*n* = 20 and *n* = 10, respectively). For our analyses, we developed a convolutional neural network (CNN) for automatic muscle segmentation using the baseline scans from these two studies with an additional 24 participants from a third study (16 with idiopathic neck pain and nine controls). This resulted in 83 unique participant baseline scans, of which 70 were used for the training dataset, and 13 that were manually traced by two raters were used for the testing dataset. All participants provided written informed consent, and the studies were approved by the University of Newcastle Human Research Ethics Committee (H-2014-0416, H-2014-0233, and H-2015-0235). The two intervention trials were registered with the Australian New Zealand Clinical Trials Registry (physiotherapy: ANZCTR12614000303640; chiropractic: ACTRN12615000256572).

Participants were recruited from the general community using advertising (paper and electronic notices, social media, and a local research volunteer register and newsletter) and were screened for eligibility by telephone. Eligible participants were aged between 18 and 55 years. Those with neck pain were eligible if their current neck pain was ≥4 out of 10 on a verbal numerical pain rating scale and if their pain interfered with daily activity at least “moderately’ over the previous four weeks (question 5 asked verbally from the 12-Item Short-Form Health Survey [31]). They were excluded if headache or dizziness was their primary complaint (though we did not exclude those with occasional headaches related to their neck pain) or if they had (a history of) migraine headaches, trauma/surgery to the neck, diabetes, peripheral vascular disease, inflammatory disease, neurologic conditions, neuropathic pain (score of  10 on the Self-Reported Leeds Assessment of Neuropathic Symptoms and Signs [32]) referred symptoms past the tip of the shoulder, receiving workers’ compensation, history of long-term steroid use, currently taking anticoagulant medication, or were pregnant/breastfeeding. (Additional participants included in the sample used to develop the CNN were not excluded on the basis of referred symptoms past the tip of the shoulder, and nine of these additional 24 participants had mild radiculopathy.) Asymptomatic participants were excluded if they had any current musculoskeletal pain in any area, if they had sought treatment for neck pain in the previous two years, or if they could not be matched to a participant with neck pain by sex and age within 5 years. All participants needed to be able to undergo an MRI exam (no metallic implants, pacemakers, claustrophobia, not pregnancy).

Participants with pain were randomised to receive various interventions: manual therapy and exercise [33,34] with or without task-specific training [35] (physiotherapy study), manual manipulation and exercise or wait-list control (chiropractic study [36]). All intervention participants were assigned to attend six sessions, 45 min in length, once per week for six weeks following their baseline MRI. In the physiotherapy study, interventions included manual therapy consisting of joint mobilisations as described by Maitland et al. [37] to cervical and upper thoracic joints as indicated and individually tailored exercises that included range of motion and stretching to the cervical and/or thoracic spine, postural training and deep cervical flexor training as described by Jull et al. [38,39]. In the chiropractic study [36], intervention participants received high-velocity, low-amplitude thrust manipulations as indicated in the upper thoracic vertebrae using a supine procedure [40] and in the cervical spine using the Gonstead Cervical Chair procedure (described by Bergman and Peterson as the “seated index/pillar push” [40]), with tailored dynamic neuromuscular stabilisation exercises [41], trigger point work, stretching, posture education and task modification. In each study, a single professional performed the interventions for both groups. Participants were not blinded to the treatment they received, though all interventions were described as ‘expected to relieve neck pain’.

At six months, participants with pain completed the Global Rating of Change Score on an 11-point scale by answering the question, “With respect to your neck pain, how would you describe yourself now compared to before you had the intervention on your neck?” The 11-point GROC scale was anchored by ‘very much worse (−5)’ on the left and ‘completely recovered (+5)’ on the right, with ‘unchanged (0)’ in the middle of the scale [42]. A score of 3 points or more was considered “recovered”; ≤2 was classified as “not recovered”.

The following variables contextualised the participant sample: age, sex (male/female), weight (kg using a standard scale: Seca, Model 7621019009), height (cm using a standard stadiometer), body mass index (BMI), physical activity level (Godin Shepherd Leisure-time Physical Activity Questionnaire [43]), and depressive symptoms (Center for Epidemiologic Studies Short Depression Scale [CES-D 10] [44]). In participants with neck pain, we also collected neck disability (Neck Disability Index, NDI [45]), pain duration (months) and pain intensity (100 mm visual analogue scale [VAS] anchored by ‘no pain’ on the left and ‘worst pain imaginable’ on the right for current, past 24 h and past four weeks on average [46]). All participants had their neck range of motion measured in flexion, extension and rotation (right and left), using the Cervical Range of Motion instrument (CROM, Performance Attainment Associates, Minnesota, IL, USA [47]), recording the average of three repetitions.

**Figure 1:**
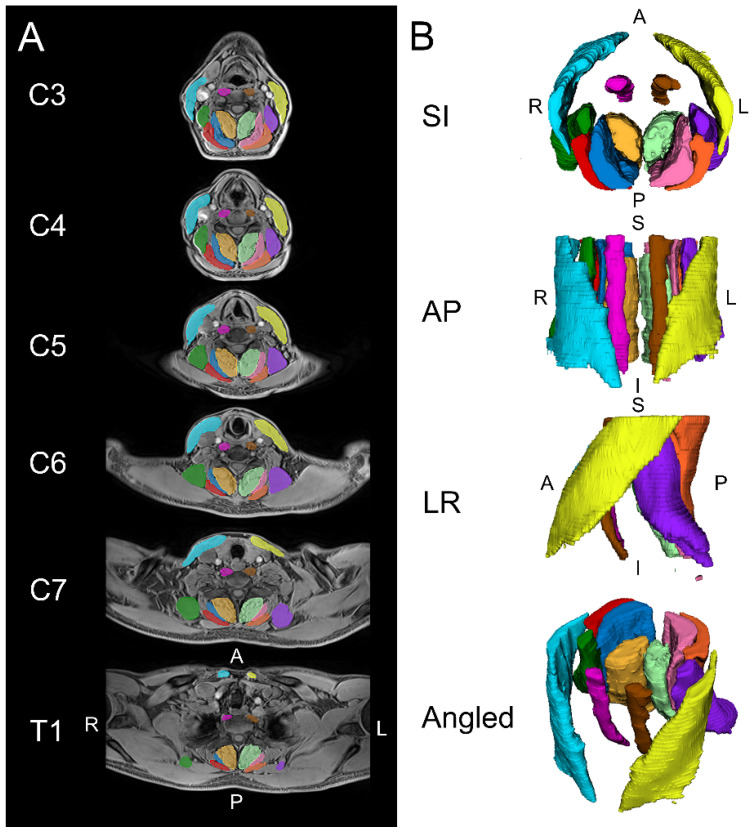
Example images are shown for the automated segmentation of the cervical spine muscles from C3 to T1 using a convolutional neural network (CNN). (**A**) Two-dimensional axial slices displaying the segmentations over the water image. (**B**) Three-dimensional renderings of the cervical spine muscle segmentations. The muscle groups segmented included the levator scapula (left  =  purple, right  =  dark green), multifidus (left  =  light green, right  =  amber), semispinalis capitis (left  = pink, right  =  dark blue), splenius capitis (left  =  orange, right  =  red), longus colli (left  =  brown, right  =  magenta), and sternocleidomastoid (left  =  yellow, right  =  light blue). S = superior, I = inferior, A = anterior, P = posterior, L = left, R = right.

MFI, muscle volume, and relative volume were measured from MR images from the intervertebral disc of C2/3 through the intervertebral disc of T1/2 (Figure 1). MRI was performed on a Siemens Magnetom Prisma 3-tesla scanner utilising a 64-channel head/neck array coil. An axial, VIBE (T1-weighted gradient echo) using two-point Dixon technique (Dixon-VIBE) (TR/TE1/TE2 7.05/2.46/3.69 ms) was acquired with a 320 × 320 mm field of view and 448 × 448 acquisition matrix (0.7 mm in-plane resolution) with a slice thickness of 3 mm. A single slab with 52 slices was acquired from the cephalad portion of C3 through the caudal portion of the T2 vertebral end plate in 6:23 min. Axial slices were aligned parallel to the C2/3 intervertebral disc allowing MRI slices to perpendicularly intersect muscles. The participant’s head was positioned in an approximately neutral position, using the same coil for every study to standardise alignment. A foam pad was placed under the head of the participant for comfort, while additional padding was placed on either side of the head to minimise head movement. The participant was instructed to remain stationary throughout the examination.

To identify each axial slice in relation to the cervical vertebrae, we used a sagittal localiser view to assign individual slices to vertebral levels. Firstly, the slices closest to the midsection of each intervertebral disc were identified. These identified the disc space and were assigned to the spinal level cephalad of the disc. Lastly, the slices between those that identified disc spaces were assigned to the appropriate spinal level.

Two blinded raters (*SS*, *OK*) manually segmented the muscles of interest (i.e., left and right levator scapulae, multifidus including semispinalis cervicis (MFSS), semispinalis capitis, splenius capitis including splenius cervicis (SCSC), longus colli and sternocleidomastoid) across the C1–T1 cervical region on all images taken at baseline using anatomical cross-references [12] as previously described [26,27,48]. Both raters had training in cervical spine anatomy and interpreting the muscle boundaries from the MR images.

To develop the CNN, all baseline images that had been manually segmented were assigned to a training dataset (*n* = 70; 45% female; mean age 35.9 years, SD 10.4; BMI 25.1 kg/m^2^, SD 4.1), except the images from 13 participants manually segmented by two raters that were assigned to a testing dataset (*n* = 26; 62% female; age 38.6, 10.5; BMI 27.9, 5.8). We trained a modified 2D U-Net CNN for image segmentation using the MONAI framework for deep learning in healthcare imaging [49,50]. A NVIDIA RTX 3090 24GB graphical processing unit (GPU, NVIDIA, Santa Clara, CA, USA) (spatial window batch size = 10, batch size = 1, optimiser = AdamW, loss function = DiceCEloss, weight decay = 0.0001, and learning rate = 0.001) was used for model training. The 2D CNN model was trained on axial slices of the water images using a spatial window size of 368 × 144 × 1. The CNN used in this study is available, open-source, at https://github.com/MuscleMap/MuscleMap (accessed on 20 June 2024). Subsequent to the development of the CNN, all muscle data used in the current study were extracted using the CNN, including data from baseline images.

### Data Analysis

As this was a sample of convenience, the sample size was not calculated a priori. CNN segmentation performance was evaluated by comparing its output to the ‘ground truth’, defined as the mean of the values extracted from the manual segmentations of both blinded raters on the testing dataset (*n* = 13). CNN segmentation accuracy and reliability were assessed using segmentation metrics (e.g., Sørensen–Dice index), intraclass correlation coefficients (ICC_2,1_), correlation plots and Bland–Altman analyses in the testing dataset (*n*  =  13) using previously described methods [48]. Similarly, we also assessed the interrater reliability of manual segmentation between the two raters. ICCs were interpreted as <0.40 poor, 0.40–0.59 fair, 0.60–0.74 good, and 0.70–1.00 excellent [51]. See Supplementary Table S1 for descriptions of each of the segmentation metrics calculated.

Participant characteristics at baseline were analysed with descriptive statistics, with potential baseline differences between groups determined using one-way analysis of variance with post-hoc tests adjusted using the least significant difference (for continuous measures) and Pearson’s Chi-square for categorical measures. Participant characteristics were analysed including the asymptomatic group, except for variables not measured in the asymptomatic group and for range of motion, where the asymptomatic group would be expected to be different at baseline.

Bonferroni-adjusted estimated marginal means (EMM) from linear mixed regression models for each of MFI, volume and relative volume are used to report group means (SD), determine changes between baseline and 6 months, and determine between-group differences at baseline and at 6 months accounting for participant group, side (left/right), spinal level, age, sex, body mass index, time (baseline vs. 6 months), and interaction for group × time. Models were conducted for each of the six muscle/muscle groups separately and for all muscles together. As models were analysed by MRI slice, and each participant had data from multiple slices and time points, we included a random effect for the participant in all models. For each model, the assumptions of normality, linearity, homoscedasticity, and independence of residuals were checked and confirmed for each model. *p*-values of 0.05 were considered statistically significant.

All differences between time points or between groups are reported as outputs from the post-hoc tests of the regression models; therefore, reported values are adjusted for age, sex, BMI, spinal level, and side. Within-group changes at six months are adjusted for baseline values. Between-group comparisons account for the two time points. Mixed models were completed using IBM SPSS Statistics, Version 28.0 (Armonk, NY, USA: IBM Corp).

## 3. Results

### 3.1. Participants

Participants with neck pain were recruited from May through December 2015, with asymptomatic matched controls subsequently recruited through May 2017. Of 151 volunteers with neck pain screened, 33 enrolled (22 in the physiotherapy study and 11 in the chiropractic study). Reasons for exclusion were previous trauma (e.g., motor vehicle collision) or surgery (29%, *n* = 43), migraines (15%, *n* = 22), did not meet pain criteria, usually with pain levels too low (12%, *n* = 18), age > 55 years (12%, *n* = 17), radiculopathy (9%, *n* = 14), declined participation or not contactable after inquiring about the study (7%, *n* = 10), neuropathic pain or fibromyalgia (5%, *n* = 8), reports of dizziness of unknown origin (2%, *n* = 3), currently receiving treatment (1%, *n* = 2), congenital fused vertebrae (1%, *n* = 1), diabetes (1%, *n* = 1), or reason not recorded (8%, *n* = 12). Two participants were excluded from the intervention studies after their baseline scan (one had an unrelated injury, and the other was deemed not eligible for the assigned intervention by the treating practitioner); their baseline MRI scans are included in the data used to develop the CNN. Asymptomatic volunteers were enrolled when their age was within 5 years, and their sex matched a pain participant.

Twenty-one participants with neck pain enrolled in the physiotherapy study and were randomised to tailored manual therapy + exercise with or without task specific training; 10 enrolled in the chiropractic study and were randomised to manipulation + exercise or no treatment control. Seven participants with pain are missing their GROC score at six months (six did not return, and one did not complete questionnaires); thus, their data are removed from the current analyses, as they could not be categorised into an outcome group. Seven asymptomatic participants did not return at six months; their baseline scan is included. Characteristics of the included participants are reported in Table 1. Groups were not significantly different in terms of the characteristics listed in Table 1, and these characteristics did not significantly differ for the missing pain participants (*n* = 7) compared to the two pain groups (Table 1).

### 3.2. CNN Performance

The two-dimensional CNN model training was completed in 30,000 iterations. CNN segmentation accuracy was good to excellent with Sørensen–Dice ≥ 0.73 (range 0.73 to 0.87). We report good to excellent CNN reliability for MFI with ICC_2,1_ ≥ 0.708 (range 0.708 to 0.977) except for the right SCM (ICC_2,1_ = 0.565), left SCM (ICC_2,1_ = 0.482), and the right longus colli (ICC_2,1_ = 0.708). CNN reliability was excellent for muscle volume of all muscles with ICC_2,1_ ≥ 0.880 (range 0.880 to 0.973). In comparing manual segmentation between the two raters, good to excellent interrater segmentation accuracy with Sørensen-Dice ≥ 0.72 (range 0.72 to 0.87) was observed. We report good to excellent interrater reliability for MFI with ICC_2,1_ ≥ 0.633 (range 0.633 to 0.957) except for the right SCM (ICC_2,1_ = 0.454) and left SCM (ICC_2,1_ = 0.388). Interrater reliability was good to excellent for muscle volume of the individual muscles with ICC_2,1_ ≥ 0.669 (range 0.669 to 0.975). Tables and Bland–Altman and correlations plots summarizing the CNN and interrater segmentation accuracy and reliability are provided in the Supplementary Materials.

### 3.3. Changes in Muscle Composition Over Time

**Figure 2:**
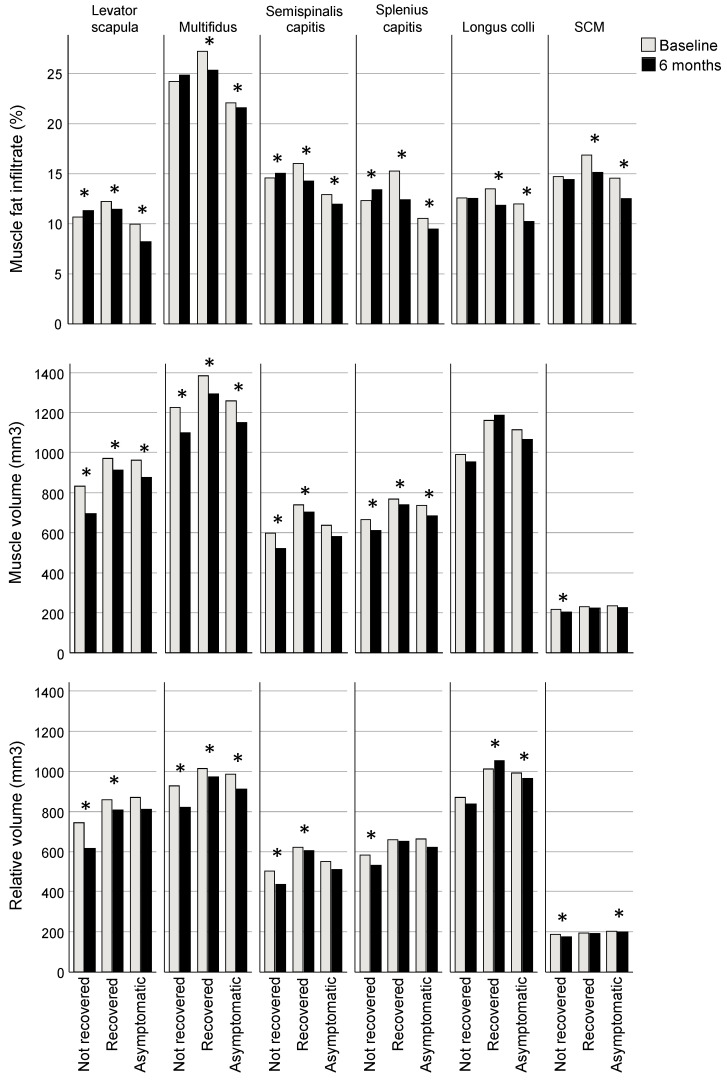
Bonferroni-adjusted estimated marginal mean differences between baseline and six months for muscle fat infiltrate (MFI), volume and relative volume. * Indicates a significant difference between baseline and six months (for MFI *p* ≤ 0.01, volume ≤ 0.032, relative volume ≤ 0.04), adjusted for age, sex, body mass index, spinal level, side (left/right), group, and interaction for group × time.

The recovered and asymptomatic groups had reduced MFI at six months compared to baseline (estimated marginal mean [EMM] difference, all muscles analysed together: recovered −1.6%; 95% CI −1.8, −1.4; asymptomatic −1.6; −1.9, −1.4; *p* < 0.001) whereas the group classified as not recovered had increased MFI compared to baseline (0.4; 0.1, 0.7; *p* = 0.014). Consistent across regression models for individual muscles, each muscle in the recovered and asymptomatic groups had significantly less MFI at 6 months compared to baseline (EMM differences ranging from −0.5 to −3.0% for recovered and −1.3 to −2.1% for asymptomatic groups, *p* < 0.001 for all muscles except levator scapula *p* = 0.012; Table 2, Figure 2). Three muscles in the not recovered group had greater MFI at six months (levator scapula, semispinalis capitis, SCSC, with EMM differences ranging from 0.5 to 1.0%; *p* ≤ 0.009; Table 2, Figure 2).

Volume was less for all groups at 6 months compared to baseline (EMM difference, all muscles analysed together: not recovered −37.3 mm^3^, 95% CI −57.3, −17.2, *p* < 0.001; recovered −31.7; −50.5, −13.0, *p* < 0.001; asymptomatic −14.7; −27.3, −2.1; *p* = 0.022). Examining regression models for individual muscles, volume was reduced in the recovered group at 6 months in all of the extensor muscles (EMM differences ranging from −112.5 mm^3^ [95% CI −137.0, −88.0] for MFSS to −26.2 [−39.5, −12.9] for SCSC; *p* ≤ 0.001), though the not recovered group also demonstrated reduced volume in extensors (ranging from −101.8 [−130.6, −73.0] for levator scapulae to −20.1 [−34.3, −5.9] for SCSC; *p* ≤ 0.032) and the SCM (−14.1 [−19.6, −8.7]; *p* < 0.001), and the asymptomatic group had reduced volume in levator scapulae (−33.5 [−51.5, −15.5]) and MFSS (−43.9 [−60.4, −27.5]); *p* < 0.001) (Table 2, Figure 2).

Relative volume was reduced at 6 months compared to baseline in the not recovered group (EMM difference, all muscles analysed together −32.5 mm^3^; 95% CI −49.5, −15.5; *p* < 0.001) but not significantly different for the recovered (−15.0; −30.9, 0.8; *p* = 0.063) and asymptomatic groups (1.0; −10.1, 11.3; *p* = 0.913). For analyses of individual muscles, relative volume was reduced at six months for all muscles in the not recovered group except for longus colli (EMM differences ranging from −12.1 mm^3^ [95% CI −17.0, −7.2] for the SCM to 95.1 [−121.4, −68.9] for the levator scapula; *p* ≤ 0.023), reduced for three muscles in the recovered group (levator scapula (−66.5 [−90.8, −42.1]), MFSS (−64.1 [−82.8, −45.3]), and semispinalis capitis (−16.9 [−32.7, −1.1]; *p* ≤ 0.036), and in the asymptomatic group, reduced for one muscle (MFSS (−18.3 [−30.9, −5.7]; *p* = 0.005) and greater for another (longus colli (16.0 [0.7, 31.4]; *p* = 0.040) (Table 2, Figure 2).

### 3.4. Differences between Groups

At baseline, the recovered group had greater MFI than the asymptomatic group (EMM difference, all muscles analysed together 3.5%, 95% CI 0.2, 6.8; *p* = 0.036). There were no other significant between-group differences at baseline or six months in EMMs when analysing all muscles together and accounting for all confounders. Examining the regression models for individual muscles, at baseline, the recovered group had greater MFI compared to the asymptomatic group in the MFSS (EMM difference 5.4%; 95% CI 1.5, 9.3; *p* = 0.004), semispinalis capitis (3.4; 0.2, 6.7; *p* = 0.037), and SCSC (5.5; 1.9, 9.1; *p* = 0.001).

There were no other significant differences between groups at baseline from the post-hoc tests of the regression models for individual muscles. At 6 months, the recovered group had greater MFI than the asymptomatic group in the levator scapula (EMM difference 4.1% [0.5, 7.7]; *p* = 0.021), semispinalis capitis (3.3 [0.02, 6.6]; *p* = 0.048) and SCSC (3.9 [0.3, 7.5]; *p* = 0.030), and less relative volume in the levator scapula (−130.1 [−252.7, −7.4]; *p* = 0.034) (Table 2).

## 4. Discussion

This study investigated the relationship between cervical muscle composition on MRI and chronic idiopathic neck pain over a six-month period. Individuals defined as recovered at 6 months (GROC ≥ 3) had less MFI compared to baseline in all muscles, whereas those who were not recovered (GROC ≤ 2) had greater MFI in three of the six muscles investigated (levator scapula, semispinalis capitis and SCSC). For the recovered group, the relative volume increased with no change in overall volume for longus colli, suggesting those who recovered may have increased their muscle mass particularly in longus colli. At six months in the group that did not recover, muscles with greater MFI (levator scapula, MFSS and SCSC) had less volume and relative volume compared to baseline, suggesting MFI may have accumulated over time occupying the muscle space. These changes in muscle composition may reduce the capacity to generate or sustain muscle forces, which may affect neck position, leading to anatomical changes that might underpin pain chronicity. There were few between-group differences in muscle composition, though the recovered group had greater MFI than the asymptomatic group in 3 of 6 muscles investigated at baseline and 6 months. Decreased MFI in the recovered group and increased MFI in the not recovered group over time suggest that changes in MFI are related to recovery from idiopathic neck pain. As participants with pain received neck muscle exercise interventions, this relationship between neck pain recovery and MFI reduction suggests a potential mechanism supporting targeted neck muscle training [34].

The majority of participants with neck pain in the current study received a form of treatment that included exercise, with many having deep cervical muscle flexion [34,39] as part of their treatment program. Changes in muscle composition (that were not reported to the patient) represent a possible mechanism for self-reported improvement, as reduced cervical MFI was observed in the recovered group: those for whom this treatment approach was successful. Specifically, longus colli was observed to have greater relative volume alongside reduced MFI at six months in the recovered group, suggesting an increase in active functional muscle mass. In contrast, other muscles showed reductions in MFI but no changes in relative volume. Longus colli is a muscle that is specifically activated with the deep neck flexor exercise that many participants received [52], suggesting the intervention was successful in affecting the target mechanism. Future research might develop a decision tree to identify patients who may benefit from mechanically focused exercise intervention (perhaps by identifying those with high MFI) and establish a specific intervention approach that includes deep cervical flexor training. If this approach is shown to be effective for reducing symptoms and MFI, it may lead to recommendations for interventions that target MFI and enable MFI to be used as a biomarker for theragnosis and prognosis.

At 6 months follow-up, the recovered group had less MFI compared to the baseline, whereas the not recovered group had the same or more than their baseline values. Reduced MFI in the recovered group associated with self-reported recovery following specific muscle training suggests that the underlying pain mechanism in the recovered group may have been predominantly mechanical or nociceptive [53], as this would support the assumption of a successful outcome from targeted muscle training and manual therapy. The lack of self-reported recovery and minimally changed MFI in the not recovered group suggests their underlying predominant pain mechanism was unlikely mechanical or nociceptive. Their predominant pain mechanism may have been driven by centrally mediated mechanisms. These findings suggest MFI might be a useful biomarker to predict those who might respond to mechanical treatment focused on muscles, whereas those without excessive MFI might require other interventions, such as psychologically-informed interventions [54]. Current AI-based studies, using larger numbers of participants, are underway and aimed to establish normative reference values for MFI controlled by age, sex, gender, race, and ethnicity. The normative reference dataset of muscle composition across the lifespan will help diagnose pathology, gauge the efficacy of interventions, and develop new outcome measures capable of accurately assessing the impact of change in muscle composition (https://github.com/MuscleMap/MuscleMap, accessed on 20 June 2024).

Unexpectedly, there were few differences in muscle composition between asymptomatic, recovered or not recovered groups at baseline or six months. This might suggest that when individual characteristics are accounted for in a mixed model (i.e., age, sex and BMI), differences between individuals overshadow any group differences [55,56], and thus group differences are not detected. Regarding between-group differences, the recovered group had greater MFI than the asymptomatic group at baseline in levator scapula, semispinalis capitis and SCSC. Despite MFI reduction for those who recovered, the group mean MFI for the recovered group remained greater than the asymptomatic group at six months.

Notably, all of the relationships regarding muscle composition in this study have been analysed with adjustment for age, sex, BMI, spinal level, and side (left/right) in regression models. The models showed that muscle composition for all variables and muscles differed significantly with spinal level (patterns of differences depended on the muscle examined and were related to the muscle anatomy) and side (right side typically slightly greater), so these factors were included in all models. Older age was associated with greater MFI in all cervical muscles and less relative volume (active muscle mass) in MFSS, SCSC and SCM, suggesting that MFI may increase with age, reducing the available muscle mass for head and neck control. The relationship between increased MFI and increased age has been confirmed in multiple studies in asymptomatic individuals in the lumbar spine [57,58,59,60,61,62,63,64] and triceps surae [65] and in individuals with degenerative cervical myelopathy [66,67]. Sex was not associated with MFI, though males had greater volume and relative volume than females likely due to larger muscles as a result of anthropometrics; males as a group tended to be taller and weigh more. Greater BMI was associated with greater MFI for all cervical muscles studied. However, greater BMI was associated with greater volume and relative volume for only two muscles: the levator scapula and the SCM. Associations between BMI and MFI are likely related to more body fat overall, though the relationship between MFI and self-reported recovery remained when accounting for BMI in the models. As age, sex and BMI were strongly associated with muscle composition for many muscles, they were retained in the models analysing each muscle. These findings are consistent with previous studies in the cervical spine [26,27,68].

The clinical implications of our findings may suggest that muscle composition may change in response to therapies, particularly if the primary pain mechanism is mechanical or nociceptive. All participants with pain received mechanically-focused interventions (manual therapy and exercise), suggesting that those who responded to this therapy regime and recovered might have had more of a mechanical or nociceptive pain mechanism (as compared to a neuropathic or central pain mechanism) [53]. If that hypothesis is accepted, then it may support investigations to determine if MFI may be a possible biomarker to identify patients who may benefit from manual therapy and exercise, that is, patients with a pain mechanism that is predominantly mechanical or nociceptive.

This study has several limitations. First, the use of GROC to classify recovery is limited, although it is commonly used in clinical research [42] and recommended by research consortia [69]. Defining a GROC cut-off score to classify ‘recovery’ is somewhat arbitrary. The minimally clinically important change has been reported as 2, 2.5 or 3 [42,70,71], with higher cut-offs shown to better distinguish between improved and not improved patients [72]. Further, patients with less severe symptoms at baseline (like the participants in the current study) typically report smaller change scores [73]; thus, the cut-off score of ≥3 in the current study increases the certainty of true improvement for those assigned to the recovered group. The number of participants in each of the pain groups was relatively small, so there was inadequate statistical power to investigate the effects of the different treatment approaches. Participants in the current study had a lengthy history of neck pain (92% had pain ≥ 1 year and 58% had pain ≥ 5 years, Table 1). It is unknown whether MFI might develop rapidly at the onset of pain, as in whiplash-associated disorder [20], or if it is present prior to pain onset.

Our results linking reduced MFI with recovery should be viewed with caution. Changes in MFI are unlikely to be a cause of changes in pain and are only one possible physiological mechanism that may occur alongside pain changes. It is likely that reductions in pain severity are the result of complex interactions of multiple factors, such as psychological (e.g., cognitions and emotions) and social (e.g., socioeconomic and cultural) systems [74]. These factors were beyond the scope of this study but warrant examination in future studies that fully characterize these interactions before and after the exercise program. Nevertheless, improvement in clinical function following reduced muscle fat infiltration might be expected through increased muscle functional capacity per unit size [75]. It has been hypothesized that this will improve spinal resilience and/or reduce muscle fatiguability to metabolic demands [74,76]. Future research is warranted to further elaborate the inter-related associations between muscle composition, muscle function and spinal pain. Moreover, longitudinal studies with larger samples are needed to better understand the causal relationship between MFI, head and neck control, and pain. Automated methods of classifying muscle composition [77], as used in the current study, and/or data sharing will be necessary to potentially uncover findings that are masked in smaller samples due to variability between participants. Data sharing will require consideration of ethical and privacy issues [78,79].

## 5. Conclusions

This study investigated 31 participants with chronic idiopathic neck pain and 30 controls over six months. Reduced cervical MFI was related to self-reported recovery from chronic neck pain, accounting for age, sex, BMI, spinal level, and side (left/right). The 12 participants who recovered had greater MFI at baseline and greater reductions in MFI over six months compared to the 12 participants who did not recover. Thus, MFI may be a possible biomarker to identify patients expected to recover following intervention.

## Figures and Tables

**Table 1:** Baseline characteristics of participants.

Characteristic	Not Recovered (*n* = 12)	Recovered (*n* = 12)	Asymptomatic (*n* = 30)	Pain Not Categorised (Missing) *n* = 7	*p* Value *
Age (yr), mean (SD)	35.3 (10.7)	32.4 (8.5)	34.3 (11.0)	38.7 (10.5)	≥0.211
Sex (female), number (%)	7 (58)	2 (17)	13 (43)	2 (29)	0.176
Weight (kg), mean (SD)	74.0 (18.4)	82.6 (13.0)	74.3 (17.2)	82.9 (7.1)	≥0.132
Height (cm), mean (SD)	171.7 (10.6)	179.1 (8.3)	169.2 (10.7)	177.0 (13.9)	≥0.009 ^‡^
BMI (kg/m^2^), mean (SD)	24.8 (4.8)	25.8 (3.8)	25.9 (4.2)	26.8 (4.8)	≥0.403
Physical activity (category), number (%)					0.919
Insufficiently active	4 (33)	3 (25)	5 (17)	1 (11)	
Moderately active	1 (8)	2 (17)	6 (20)	1 (11)	
Active	7 (58)	7 (58)	19 (63)	4 (57)	
CES-D 10 ^†^ (category), number (%)					0.059
Depressive symptoms (CES-D ≥ 10 points)	3 (25)	3 (25)	2 (7)	3 (43)	
Not depressed (CES-D < 10 points)	9 (75)	9 (75)	28 (93)	3 (43)	
Pain, 0–100 mm visual analogue scale (mm), mean (SD)					
Current	30.7 (20.4)	29.3 (15.6)	–	30.4 (18.4)	≥0.859
24 h recall	36.8 (20.9)	40.0 (13.3)	–	36.4 (24.6)	≥0.683
4-week recall	42.6 (14.8)	41.2 (15.3)	–	41.6 (27.5)	≥0.861
Neck Disability Index (0–50), mean (SD)	13.3 (4.6)	12.6 (2.9)	–	13.8 (6.3)	≥0.574
Duration of neck pain (months), mean (SD)	82.5 (37.6)	52.0 (40.2)	–	56.3 (61.6)	≥0.106
Duration of neck pain (category), number (%)					0.252
3 to 12 months	0 (0)	2 (17)	–	1 (14)	
1 to 5 years	3 (25)	5 (42)	–	4 (57)	
5 + years	9 (75)	5 (42)	–	2 (29)	
Neck flexion range of motion [ROM] (°), mean (SD)	49.6 (8.3)	56.4 (9.2)	60.7 (7.9)	46.6 (6.2)	0.020 ^§^
Neck extension ROM (°), mean (SD)	60.9 (9.4)	58.5 (15.0)	65.7 (12.3)	53.9 (16.7)	≥0.286
Neck right rotation ROM (°), mean (SD)	60.6 (10.1)	61.2 (8.1)	68.6 (6.5)	61.5 (12.4)	≥0.849
Neck left rotation ROM (°), mean (SD)	62.1 (10.6)	60.8 (8.2)	67.4 (5.7)	57.9 (11.9)	≥0.380

* *p*-values for comparison of groups at baseline. For continuous measures, the *p*-value listed is the smallest of the *p*-values for the ANOVA post hoc tests (least significant difference) comparing pairs of groups. For categorical measures, the *p*-value is the overall Pearson’s Chi-square. ^†^ Center for Epidemiologic Studies Short Depression Scale. ^‡^ Recovered group was taller than the asymptomatic group (mean difference 9.9 cm, 95% CI 2.6, 17.1); no other differences in height between groups (*p* ≥ 0.084). ^§^ For ROM, ANOVAs were performed excluding the asymptomatic group, which would be expected to be different at baseline. The recovered group had greater baseline flexion ROM than the Pain not categorised (missing) group (mean difference 9.7°, 95% CI 1.7, 17.8); no other between-group differences in flexion ROM (*p* ≥ 0.055).

**Table 2:** Bonferroni-adjusted estimated marginal means (EMM) and mean differences (95% CI) from linear mixed regression models for each muscle for muscle fat infiltrate (MFI), volume and relative volume, accounting for age, sex, body mass index, spinal level, side (left/right), and interaction for group × time.

Muscle	Groups							Difference within Groups				Difference between Groups		
	Baseline			6 Months				6 Months Minus Baseline				At 6 Months		
	Not Rec (*n* = 12)	Rec (*n* = 12)	Asymp (*n* = 30)	Not Rec (*n* = 12)	Rec (*n* = 12)	Asymp (*n* = 23)		Not Rec (*n* = 12)	Rec (*n* = 12)	Asymp (*n* = 30)		Not Rec minus Asymp	Rec minus Asymp	Not Rec minus Rec
MFI (%)														
Levator scapula	10.2 (7.7, 12.7)	13.1 (10.5, 15.6)	10.5 (8.9, 12.0)	10.7 (8.2, 13.2)	12.6 (10.1, 15.1)	8.5 (7.0, 10.1)		0.5 ** (0.1, 0.9)	−0.5 * (−0.9, −0.1)	−2.0 * (−2.2, −1.7)		2.2 (−1.5, 5.9)	4.1 * (0.5, 7.7)	−1.9 (−6.3, 2.6)
Multifidus with semispinalis cervicis	24.8 (22.1, 27.5)	28.4 (25.7, 31.2)	23.1 (21.4, 24.7)	24.6 (21.9, 27.5)	26.9 (24.1, 29.6)	21.8 (20.1, 23.4)		−0.2 (−0.8, 0.4)	−1.6 *** (−2.2, −1.0)	−1.3 *** (−1.7, −0.9)		2.8 (−1.1, 6.8)	5.1 (1.2, 9.0)	−2.3 (−7.1, 2.5)
Semispinalis capitis	14.6 (12.3, 16.9)	16.6 (14.2, 18.9)	13.1 (11.7, 14.5)	15.3 (13.0, 17.6)	15.0 (12.7, 17.4)	11.8 (10.3, 13.2)		0.7 ** (0.3, 1.2)	−1.5 *** (−1.9, −1.1)	−1.4 *** (−1.7, −1.1)		3.5 * (0.2, 6.9)	3.3 * (0.02, 6.6)	0.2 (−3.8, 4.3)
Splenius capitis with splenius cervicis	12.1 (9.6, 14.6)	16.4 (13.8, 18.9)	10.9 (9.4, 12.5)	13.1 (10.6, 15.6)	13.3 (10.8, 15.9)	9.4 (7.9, 11.0)		1.0 *** (0.5, 1.5)	−3.0 *** (−3.5, −2.6)	−1.5 *** (−1.8, −1.2)		3.7 (−0.03, 7.3)	3.9 * (0.3, 7.5)	−0.2 (−4.7, 4.2)
Longus Colli	12.1 (9.7, 14.5)	14.3 (11.9, 16.8)	12.6 (11.1, 14.1)	11.9 (9.5, 14.3)	13.0 (10.6, 15.5)	10.5 (9.0, 12.0)		−0.2 (−0.6, 0.1)	−1.3 *** (−1.6, −1.0)	−2.1 *** (−2.3, −1.9)		1.4 (−2.1, 4.9)	2.6 (−0.9, 6.0)	−1.1 (−5.4, 3.1)
Sternocleidomastoid	14.4 (11.9, 16.8)	17.1 (14.6, 19.6)	14.7 (13.2, 16.2)	14.3 (11.9, 16.8)	15.2 (12.7, 17.7)	12.9 (11.3, 14.4)		−0.03 (−0.5, 0.4)	−1.8 *** (−2.3, −1.4)	−1.8 *** (−2.1, −1.5)		1.5 (−2.1, 5.1)	2.4 (−1.1, 5.9)	−0.9 (−5.3, 3.5)
Volume (mm^3^)														
Levator scapula	861.3 (775.3, 947.3)	837.6 (750.8, 924.5)	895.5 (842.7, 948.2)	759.5 (672.6, 846.4)	765.8 (678.9, 852.7)	862.0 (808.7, 915.2)		−101.8 *** (−130.6, −73.0)	−71.9 *** (−98.5, −45.2)	−33.5 *** (−51.5, −15.5)		−102.5 (−228.8, 23.8)	−96.2 (−219.2, 26.8)	−6.3 (−159.0, 146.4)
Multifidus with semispinalis cervicis	1193.9 (1102.5, 1285.3)	1206.5 (1114.4, 1298.8)	1146.9 (1090.8, 1203.0)	1112.4 (1020.4, 1204.5)	1094.0 (1001.6, 1186.4)	1102.9 (1046.5, 1159.4)		−81.5 *** (−107.2, −55.7)	−112.5 *** (−137.0, −88.0)	−43.9 *** (−60.4, −27.5)		9.5 (−124.3, 143.3)	−8.9 (−139.6, 121.7)	18.4 (−143.6, 180.5)
Semispinalis capitis	603.0 (541.0, 665.1)	636.2 (573.6, 698.9)	581.0 (542.9, 619.0)	581.5 (518.9, 644.1)	601.8 (539.1, 664.5)	577.6 (539.2, 615.9)		−21.5 * (−41.1, −1.8)	−34.4 *** (−52.8, −16.1)	−3.4 (−15.8, 9.0)		4.0 (−87.0, 95.0)	24.2 (−64.5, 113.0)	−20.3 (−130.3, 89.8)
Splenius capitis with splenius cervicis	673.8 (606.2, 741.4)	662.5 (594.3, 730.6)	683.1 (641.7, 724.6)	653.7 (585.9, 721.5)	636.2 (568.0, 704.4)	675.3 (633.7, 716.9)		−20.1 ** (−34.3, −5.9)	−26.2 *** (−39.5, −12.9)	−7.8 (−16.8, 1.2)		−21.6 (−120.4, 77.1)	−39.1 (−135.6, 57.5)	17.4 (−102.3, 137.2)
Longus Colli	1008.5 (914.7, 1097.1)	1047.7 (952.2, 1143.1)	1055.6 (997.6, 1113.7)	1001.9 (906.7, 1097.1)	1061.0 (965.5, 1156.4)	1051.6 (993.1, 1110.0)		−6.6 ^‡^ (−33.3, 20.1)	13.3 ^‡^ (−11.5, 38.1)	−4.1 ^‡^ (−21.0, 12.8)		−49.7 (−188.1, 88.8)	9.4 (−125.7, 144.5)	−59.1 (−226.7, 108.5)
Sternocleidomastoid	220.4 (202.7, 238.0)	212.6 (194.8, 230.5)	225.7 (214.9, 236.5)	206.2 (188.4, 224.0)	208.3 (190.5, 226.2)	224.3 (213.4, 235.2)		−14.1 *** (−19.6, −8.7)	−4.3 (−9.5, 0.8)	−1.4 (−4.8, 2.0)		−18.0 (−43.9, 7.8)	−16.0 (−41.2, 9.3)	−2.1 (−33.4, 29.2)
Relative volume (mm^3^)														
Levator scapula	772.5 (686.7, 858.3)	731.8 (645.2, 818.4)	806.0 (753.3, 858.6)	677.4 (590.8, 763.9)	665.3 (578.6, 751.9)	795.3 (742.3, 848.4)		−95.1 *** (−121.4, −68.9)	−66.5 *** (−90.8, −42.1)	−10.7 (−27.1, 5.8)		−117.9 (−243.7, 7.9)	−130.1 * (−252.7, −7.4)	12.1 (−140.1, 164.3)
Multifidus with semispinalis cervicis	896.9 (812.8, 981.0)	866.5 (781.6, 951.4)	893.3 (841.7, 944.9))	839.5 (755.0, 924.0)	802.4 (717.5, 887.3)	875.0 (823.2, 926.8)		−57.4 *** (−77.2, −37.6)	−64.1 *** (−82.8, −45.3)	−18.3 ** (−30.9, −5.7)		−35.5 (−158.3, 87.2)	−72.6 (−192.6, 47.4)	37.0 (−111.7, 185.8)
Semispinalis capitis	507.4 (450.9, 563.9)	530.2 (473.2, 587.1)	502.5 (467.9, 537.2)	487.8 (430.9, 544.7)	513.2 (456.2, 570.2)	508.8 (473.9, 543.7)		−19.6 * (−36.5, −2.7)	−16.9 * (−32.7, −1.1)	6.2 (−4.4, 16.9)		−21.0 (−103.7, 61.6)	4.5 (−76.2, 85.1)	−25.5 (−125.5, 74.6)
Splenius capitis with splenius cervicis	591.3 (529.9, 652.8)	556.8 (494.8, 618.9)	612.0 (574.3, 649.7)	572.2 (510.5, 633.9)	553.9 (491.9, 615.9)	614.5 (576.7, 652.4)		−19.1 **^§^ (−32.3, −5.9)	−2.9 ^§^ (−15.2, 9.4)	2.6 ^§^ (−5.8, 10.9)		−42.3 (−132.0, 47.4)	−60.6 (−148.4, 27.1)	18.3 (−90.5, 127.1)
Longus Colli	889.3 (803.2, 975.4)	904.1 (817.1, 991.0)	933.3 (880.4, 986.2)	885.0 (798.2, 971.7)	929.2 (842.2, 1016.2)	949.3 (896.1, 1002.6)		−4.3 ^†^ (−28.5, 19.9)	25.2 *^†^ (2.7, 47.7)	16.0 *^†^ (0.7, 31.4)		−64.4 (−190.4, 61.7)	−20.2 (−143.2, 102.9)	−44.2 (−196.8, 108.4)
Sternocleidomastoid	189.0 (172.7, 205.2)	177.3 (160.8, 193.7	193.5 (183.5, 203.4)	176.9 (160.5, 193.3)	177.3 (160.8, 193.7)	197.3 (187.3, 207.4)		−12.1 *** (−17.0, −7.2)	−0.2 (−4.7, 4.4)	3.8 * (0.8, 6.9)		−20.4 (−44.2, 3.4)	−20.0 (−43.3, 3.2)	−0.4 (−29.2, 28.4)

Abbreviations: Not Rec = Not recovered; Rec = Recovered; Asymp = Asymptomatic. * Post-hoc comparison significant at *p* < 0.001 ***, *p* < 0.01 **, or *p* < 0.05 *. ^†^ No group × time interaction, but an overall effect for Time. ^‡^ No group × time interaction nor time effects. ^§^ Group × time interaction but no overall effect for time.

## Data Availability

The original contributions presented in the study, including the data file used for analysis, are included in the Supplementary Material (Data S1); further inquiries can be directed to the corresponding author/s.

## Supplementary Materials

The following supporting information can be downloaded at https://www.mdpi.com/article/10.3390/jcm13154485/s1, Table S1. Descriptions of interrater segmentation metrics. Table S2. Interrater segmentation metrics for assessment of segmentation performance between two raters in the testing dataset (*n* = 13). Table S3. Accuracy and reliability of muscle fat infiltration (MFI, %) between two raters were assessed in the testing dataset (*n* = 13) for human-level interrater reliability. Table S4. Accuracy and reliability of muscle volume (mm^3^) between two raters were assessed in the testing dataset (*n* = 13) for human-level interrater reliability. Table S5. Performance of the convolutional neural network (CNN) model segmentations with respect to the ground truth assessed in the testing dataset (*n* = 13). Table S6. The accuracy and reliability of muscle fat infiltration (MFI) between the convolutional neural network (CNN) model and the ground truth were assessed in testing the dataset (*n* = 13). Table S7. Accuracy and reliability of muscle volume between the convolutional neural network (CNN) model and the ground truth were assessed in testing the dataset (*n* = 13). Figure S1. Interrater reliability and accuracy for muscle fat infiltration (MFI) between two raters (R1 and R2) in the testing dataset (*n* = 13), assessed by correlation and Bland-Altman plots for each muscle or muscle group. Figure S2. Interrater reliability and accuracy for muscle volume (mm3) between two raters (R1 and R2) in the testing dataset (*n* = 13), assessed by correlation and Bland-Altman plots for each muscle or muscle group. Figure S3. Interrater reliability and accuracy for muscle fat infiltrate (MFI, %) between the convolutional neural network (CNN) model and the ground truth (GT) in the testing dataset (*n* = 13), assessed by correlation and Bland-Altman plots for each muscle or muscle group. Figure S4. Interrater reliability and accuracy for muscle volume (mm^3^) between the convolutional neural network (CNN) model and the ground truth (GT) in the testing dataset (*n* = 13), assessed by correlation and Bland–Altman plots for each muscle or muscle group. Data S1: Data used for analysis (Excel).
