# Supplementary material for: Reduced Cervical Muscle Fat Infiltrate Is Associated with Self-Reported Recovery from Chronic Idiopathic Neck Pain Over Six Months: A Magnetic Resonance Imaging Longitudinal Cohort Study
Source: J Clin Med. 2024 Jul 31;13(15):4485. doi: 10.3390/jcm13154485 (PMC11312969; doi:10.3390/jcm13154485)
Supplement: Supplementary file 1 [file jcm-13-04485-s001.zip › Snodgrass_Supplementary_Materials_04_04_2024.pdf]

Supplementary Materials

Supplementary Table S1. Descriptions of interrater segmentation metrics.

Metric	Equation	Range	Meaning
Sørensen-Dice Index (DICE)	$\frac{2 \times \lvert SM \cap GT \rvert}{\lvert SM \rvert + \lvert GT \rvert}$	0 – 1	Spatial overlap between masks
Jaccard Index	$\frac{\lvert SM \cap GT \rvert}{\lvert SM \rvert + \lvert GT \rvert - \lvert SM \cap GT \rvert}$	0 – 1	Spatial overlap between masks
Conformity Coefficient	$1 - \frac{FP + FN}{TP}$	≤ 1	Ratio of incorrectly and correctly segmented voxels
True Positive Rate (TPR)	$\frac{TP}{TP + FN}$	0 – 1	Sensitivity
True Negative Rate (TNR)	$\frac{TN}{TN + FP}$	0 – 1	Specificity
Positive Predictive Value (PPV)	$\frac{TP}{TP + FP}$	0 – 1	Precision
Volume Ratio	$\frac{\lvert SM \rvert}{\lvert GT \rvert}$	≥ 0	Ratio of mask volumes

Abbreviations: SM = segmentation mask; GT = ground truth mask; TP = true positive, voxels correctly segmented; TN = true negative, voxels correctly segmented as background; FP = false positive, voxels incorrectly segmented, FN = false negative, voxels incorrectly segmented as background.

Supplementary Table S2. Interrater segmentation metrics for assessment of segmentation performance between two raters in the testing dataset (n = 13).

Muscle	Side	Dice	JI	CC	TPR	TNR	PPV	VR
Levator Scapula	L	0.86 ± 0.01	0.76 ± 0.01	0.67 ± 0.02	0.88 ± 0.01	1.00 ± 0.00	0.85 ± 0.02	1.04 ± 0.03
	R	0.84 ± 0.01	0.73 ± 0.02	0.62 ± 0.03	0.86 ± 0.01	1.00 ± 0.00	0.83 ± 0.02	1.04 ± 0.03
Multifidus	L	0.87 ± 0.01	0.77 ± 0.01	0.70 ± 0.02	0.87 ± 0.01	1.00 ± 0.00	0.88 ± 0.02	1.00 ± 0.03
	R	0.86 ± 0.01	0.76 ± 0.01	0.68 ± 0.02	0.84 ± 0.01	1.00 ± 0.00	0.89 ± 0.02	0.96 ± 0.03
Semispinalis Capitis	L	0.74 ± 0.02	0.59 ± 0.02	0.28 ± 0.06	0.82 ± 0.01	1.00 ± 0.00	0.68 ± 0.02	1.23 ± 0.05
	R	0.76 ± 0.01	0.62 ± 0.02	0.37 ± 0.05	0.79 ± 0.01	1.00 ± 0.00	0.74 ± 0.02	1.09 ± 0.04
Splenius Capitis	L	0.78 ± 0.01	0.65 ± 0.02	0.44 ± 0.04	0.81 ± 0.02	1.00 ± 0.00	0.76 ± 0.02	1.08 ± 0.04
	R	0.79 ± 0.01	0.66 ± 0.02	0.48 ± 0.04	0.81 ± 0.02	1.00 ± 0.00	0.79 ± 0.01	1.03 ± 0.03
Longus Coli	L	0.74 ± 0.01	0.59 ± 0.02	0.31 ± 0.04	0.70 ± 0.03	1.00 ± 0.00	0.80 ± 0.01	0.88 ± 0.04
	R	0.72 ± 0.02	0.56 ± 0.02	0.20 ± 0.07	0.67 ± 0.03	1.00 ± 0.00	0.79 ± 0.01	0.85 ± 0.04
SCM	L	0.86 ± 0.00	0.76 ± 0.01	0.68 ± 0.01	0.93 ± 0.01	1.00 ± 0.00	0.81 ± 0.01	1.15 ± 0.03
	R	0.87 ± 0.01	0.77 ± 0.01	0.70 ± 0.02	0.93 ± 0.01	1.00 ± 0.00	0.82 ± 0.01	1.14 ± 0.03

Abbreviations: Sørensen-Dice index (Dice), Jaccard index (JI), conformity coefficient (CC), true positive rate (TPR), true negative rate (TNR), positive predictive value (PPV), and volume ratio (VR). See Supplementary Table 1 for the calculation and interpretation of the segmentation metrics. Metrics shown = mean ± 1 standard error.

Supplementary Table S3. Accuracy and reliability of muscle fat infiltration (MFI, %) between two raters assessed in the testing dataset (n = 13) for human-level interrater reliability.

Muscle	Side	Mean	Bias	95% LA	$ICC_{2,1}$	$ICC_{2,1}$ 95% CI	$ICC_{2,1}$ p
Levator Scapula	L	11.5 ± 0.8	-2.0	-5.9 – 1.8	0.733	0.08 – 0.92	< 0.001
	R	12.7 ± 1.1	-3.2	-8.4 – 2.1	0.657	-0.03 – 0.90	< 0.001
Multifidus	L	29.0 ± 1.4	2.6	-3.0 – 8.2	0.656	0.06 – 0.89	0.001
	R	28.9 ± 1.5	3.9	-1.4 – 9.1	0.633	-0.09 – 0.90	< 0.001
Semispinalis Capitis	L	17.4 ± 1.1	-1.4	-4.9 – 2.2	0.843	0.44 – 0.95	< 0.001
	R	17.0 ± 0.9	-0.4	-2.3 – 1.5	0.952	0.85 – 0.99	< 0.001
Splenius Capitis	L	14.9 ± 1.0	0.2	-3.2 – 3.6	0.905	0.72 – 0.97	< 0.001
	R	15.5 ± 1.0	-1.0	-4.4 – 2.4	0.867	0.59 – 0.96	< 0.001
Longus Coli	L	15.4 ± 0.9	-0.3	-2.2 – 1.5	0.957	0.87 – 0.99	< 0.001
	R	17.0 ± 0.9	-0.2	-3.3 – 2.9	0.885	0.67 – 0.96	< 0.001
SCM	L	13.6 ± 0.8	-4.0	-9.4 – 1.4	0.388	-0.12 – 0.77	0.006
	R	14.9 ± 0.8	-3.8	-10.7 – 3.2	0.454	-0.09 – 0.80	0.009

Abbreviations: Mean = mean MFI ± standard error. Bias = mean difference between raters. LA = limits of agreement CI = confidence interval. p = F-test with true value = 0.

Supplementary Table S4. Accuracy and reliability of muscle volume (mm^3) between two raters assessed in the testing dataset (n = 13) for human-level interrater reliability.

Muscle	Side	Mean	Bias	95% LA	$ICC_{2,1}$	$ICC_{2,1}$ 95% CI	$ICC_{2,1}$ p
Levator Scapula	L	27.9 ± 2.5	-0.9	-6.9 – 5.1	0.947	0.84 – 0.98	< 0.001
	R	28.0 ± 2.7	-0.5	-4.7 – 3.6	0.975	0.92 – 0.99	< 0.001
Multifidus	L	36.1 ± 3.1	0.7	-6.2 – 7.5	0.948	0.84 – 0.98	< 0.001
	R	37.4 ± 3.3	2.2	-4.6 – 9.0	0.935	0.76 – 0.98	< 0.001
Semispinalis Capitis	L	17.4 ± 1.7	-3.2	-6.8 – 0.4	0.858	-0.02 – 0.97	< 0.001
	R	18.9 ± 2.0	-1.1	-5.0 – 2.8	0.948	0.82 – 0.98	< 0.001
Splenius Capitis	L	20.5 ± 2.1	-1.5	-6.7 – 3.7	0.928	0.77 – 0.98	< 0.001
	R	21.9 ± 1.9	-0.6	-4.3 – 3.1	0.964	0.89 – 0.99	< 0.001
Longus Coli	L	7.8 ± 0.5	0.9	-1.4 – 3.2	0.756	0.26 – 0.92	< 0.001
	R	8.2 ± 0.5	1.2	-1.3 – 3.8	0.669	0.07 – 0.90	0.001
SCM	L	31.6 ± 2.8	-4.9	-11.1 – 1.3	0.888	0.06 – 0.98	< 0.001
	R	33.7 ± 3.1	-4.3	-10.1 – 1.4	0.909	0.13 – 0.98	< 0.001

Abbreviations: Mean = mean volume ± standard error. Bias = mean difference between raters. LA = limits of agreement CI = confidence interval. p = F-test with true value = 0.

Supplementary Figure S1. Interrater reliability and accuracy for muscle fat infiltration (MFI) between two raters (R1 and R2) in the testing dataset (n = 13), assessed by correlation and Bland-Altman plots for each muscle or muscle group.

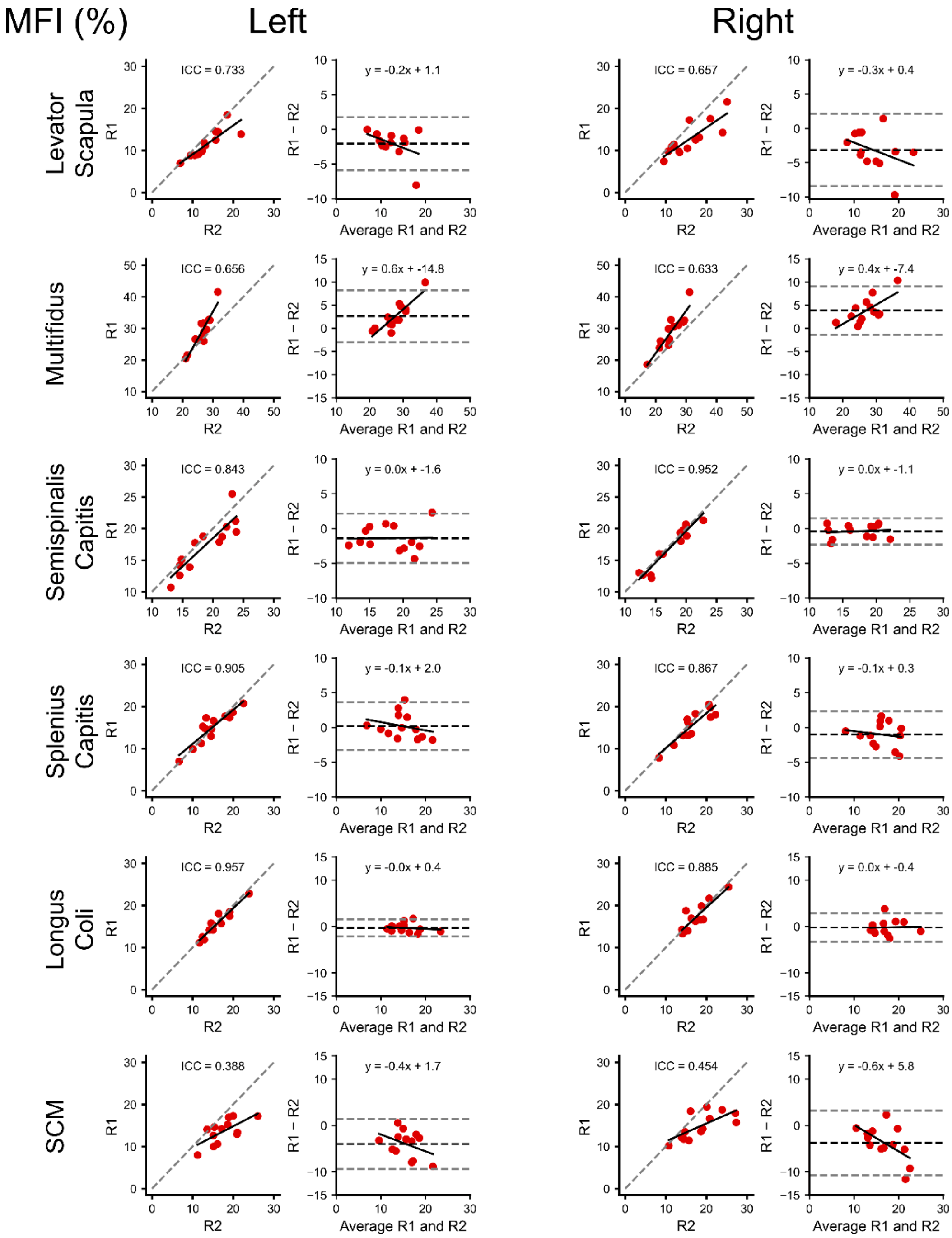

Abbreviation: ICC = intraclass correlation coefficient ($ICC_{2,1}$). In the correlation plot, the dashed gray line represents perfect correlation, and the solid black line represents the best fit line. In the Bland-Altman plots, the dashed black and gray lines indicate the mean difference (i.e., bias) ± 1.96 × standard deviation (i.e., 95% limits of agreement).

.

Supplementary Figure S2. Interrater reliability and accuracy for muscle volume (mm³) between two raters (R1 and R2) in the testing dataset (n = 13), assessed by correlation and Bland-Altman plots for each muscle or muscle group.

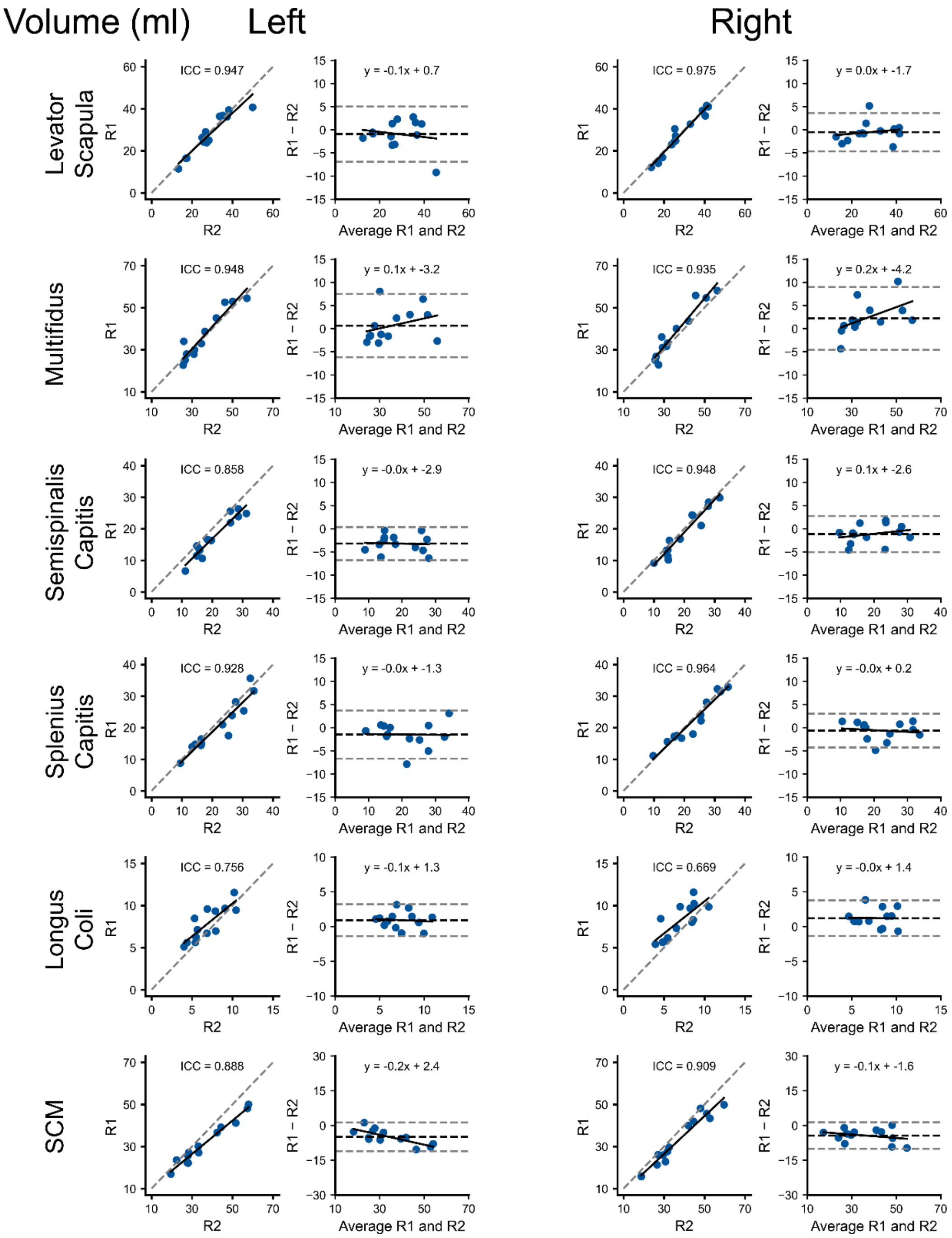

Abbreviation: ICC = intraclass correlation coefficient ($ICC_{2,1}$). In the correlation plot, the dashed gray line represents perfect correlation, and the solid black line represents the best fit line. In the Bland-Altman plots, the dashed black and gray lines indicate the mean difference (i.e., bias) ± 1.96 × standard deviation (i.e., 95% limits of agreement).

Supplementary Table S5. Performance of the convolutional neural network (CNN) model segmentations with respect to the ground truth assessed in the testing dataset (n = 13).

Muscle	Side	Dice	JI	CC	TPR	TNR	PPV	VR
Levator Scapula	L	0.82 ± 0.02	0.71 ± 0.02	0.56 ± 0.05	0.84 ± 0.02	1.00 ± 0.00	0.82 ± 0.02	1.02 ± 0.04
	R	0.83 ± 0.01	0.72 ± 0.02	0.60 ± 0.04	0.84 ± 0.02	1.00 ± 0.00	0.83 ± 0.02	1.03 ± 0.04
Multifidus	L	0.86 ± 0.01	0.76 ± 0.01	0.68 ± 0.01	0.87 ± 0.01	1.00 ± 0.00	0.86 ± 0.01	1.01 ± 0.03
	R	0.87 ± 0.00	0.76 ± 0.01	0.69 ± 0.01	0.87 ± 0.01	1.00 ± 0.00	0.87 ± 0.01	1.01 ± 0.03
Semispinalis Capitis	L	0.75 ± 0.01	0.60 ± 0.02	0.30 ± 0.05	0.76 ± 0.01	1.00 ± 0.00	0.74 ± 0.02	1.05 ± 0.04
	R	0.75 ± 0.01	0.61 ± 0.02	0.33 ± 0.05	0.76 ± 0.02	1.00 ± 0.00	0.75 ± 0.02	1.03 ± 0.03
Splenius Capitis	L	0.75 ± 0.02	0.60 ± 0.02	0.31 ± 0.06	0.74 ± 0.02	1.00 ± 0.00	0.77 ± 0.02	0.98 ± 0.04
	R	0.76 ± 0.01	0.62 ± 0.01	0.37 ± 0.03	0.77 ± 0.02	1.00 ± 0.00	0.76 ± 0.01	1.02 ± 0.03
Longus Coli	L	0.75 ± 0.02	0.60 ± 0.02	0.30 ± 0.07	0.74 ± 0.02	1.00 ± 0.00	0.76 ± 0.02	0.99 ± 0.03
	R	0.73 ± 0.02	0.58 ± 0.02	0.24 ± 0.07	0.74 ± 0.02	1.00 ± 0.00	0.73 ± 0.02	1.03 ± 0.05
SCM	L	0.84 ± 0.01	0.72 ± 0.02	0.60 ± 0.05	0.82 ± 0.03	1.00 ± 0.00	0.87 ± 0.01	0.95 ± 0.04
	R	0.85 ± 0.01	0.74 ± 0.01	0.64 ± 0.03	0.83 ± 0.02	1.00 ± 0.00	0.87 ± 0.01	0.96 ± 0.03

Abbreviations: Sørensen-Dice index (Dice), Jaccard index (JI), conformity coefficient (CC), true positive rate (TPR), true negative rate (TNR), positive predictive value (PPV), and volume ratio (VR). Metrics shown = mean ± 1 standard error.

Supplementary Table S6. Accuracy and reliability of muscle fat infiltration (MFI) between the convolutional neural network (CNN) model and the ground truth assessed in testing the dataset (n = 13).

Muscle	Side	Mean	Bias	95% LA	$ICC_{2,1}$	$ICC_{2,1}$ 95% CI	$ICC_{2,1}$ p
Levator Scapula	L	12.2 ± 0.8	-0.3	-1.6 – 0.9	0.977	0.92 – 0.99	< 0.001
	R	13.0 ± 1.0	-1.3	-3.7 – 1.1	0.909	0.42 – 0.98	< 0.001
Multifidus	L	27.8 ± 1.2	0.2	-4.5 – 4.9	0.844	0.56 – 0.95	< 0.001
	R	27.0 ± 1.5	-0.0	-5.2 – 5.1	0.865	0.61 – 0.96	< 0.001
Semispinalis Capitis	L	17.2 ± 0.9	-0.9	-3.9 – 2.1	0.887	0.63 – 0.97	< 0.001
	R	17.2 ± 0.8	0.0	-2.4 – 2.5	0.922	0.76 – 0.98	< 0.001
Splenius Capitis	L	13.6 ± 1.0	-1.2	-2.8 – 0.3	0.930	0.17 – 0.99	< 0.001
	R	15.0 ± 1.0	-1.0	-3.2 – 1.2	0.919	0.56 – 0.98	< 0.001
Longus Coli	L	14.9 ± 0.8	-0.6	-2.9 – 1.6	0.915	0.73 – 0.97	< 0.001
	R	15.3 ± 0.6	-1.8	-4.8 – 1.2	0.708	-0.00 – 0.92	< 0.001
SCM	L	13.3 ± 0.8	-2.3	-7.3 – 2.6	0.482	-0.05 – 0.81	0.010
	R	14.1 ± 0.6	-2.7	-6.8 – 1.5	0.565	-0.08 – 0.86	0.001

Abbreviations: Intraclass correlation coefficients ($ICC_{2,1}$). Mean = mean MFI ± standard error. Bias = mean difference between raters. LA = limits of agreement. CI = confidence interval. p = F-test with true value = 0.

Supplementary Table S7. Accuracy and reliability of muscle volume between the convolutional neural network (CNN) model and the ground truth assessed in testing the dataset (n = 13).

Muscle	Side	Mean	Bias	95% LA	$ICC_{2,1}$	$ICC_{2,1}$ 95% CI	$ICC_{2,1}$ p
Levator Scapula	L	28.3 ± 2.3	-0.1	-8.0 – 7.9	0.900	0.70 – 0.97	< 0.001
	R	28.5 ± 2.4	0.2	-4.8 – 5.2	0.963	0.88 – 0.99	< 0.001
Multifidus	L	35.7 ± 2.6	-0.0	-6.7 – 6.7	0.945	0.83 – 0.98	< 0.001
	R	35.9 ± 2.4	-0.3	-7.1 – 6.5	0.942	0.82 – 0.98	< 0.001
Semispinalis Capitis	L	19.5 ± 1.8	0.5	-2.6 – 3.6	0.968	0.90 – 0.99	< 0.001
	R	19.9 ± 1.9	0.5	-2.6 – 3.6	0.973	0.92 – 0.99	< 0.001
Splenius Capitis	L	20.7 ± 2.1	-0.6	-5.0 – 3.9	0.957	0.87 – 0.99	< 0.001
	R	22.3 ± 1.9	0.2	-4.3 – 4.6	0.952	0.85 – 0.98	< 0.001
Longus Coli	L	7.1 ± 0.5	-0.2	-2.0 – 1.5	0.876	0.65 – 0.96	< 0.001
	R	7.6 ± 0.5	-0.0	-1.7 – 1.7	0.892	0.68 – 0.97	< 0.001
SCM	L	31.4 ± 2.7	-2.6	-11.9 – 6.7	0.880	0.64 – 0.96	< 0.001
	R	33.9 ± 2.8	-2.0	-8.5 – 4.6	0.941	0.79 – 0.98	< 0.001

Abbreviations: Intraclass correlation coefficients ($ICC_{2,1}$). Mean = mean volume ± standard error. Bias = mean difference between raters. LA = limits of agreement. CI = confidence interval. p = F-test with true value = 0.

Supplementary Figure S3. Interrater reliability and accuracy for muscle fat infiltrate (MFI, %) between the convolutional neural network (CNN) model and the ground truth (GT) in the testing dataset (n = 13), assessed by correlation and Bland-Altman plots for each muscle or muscle group.

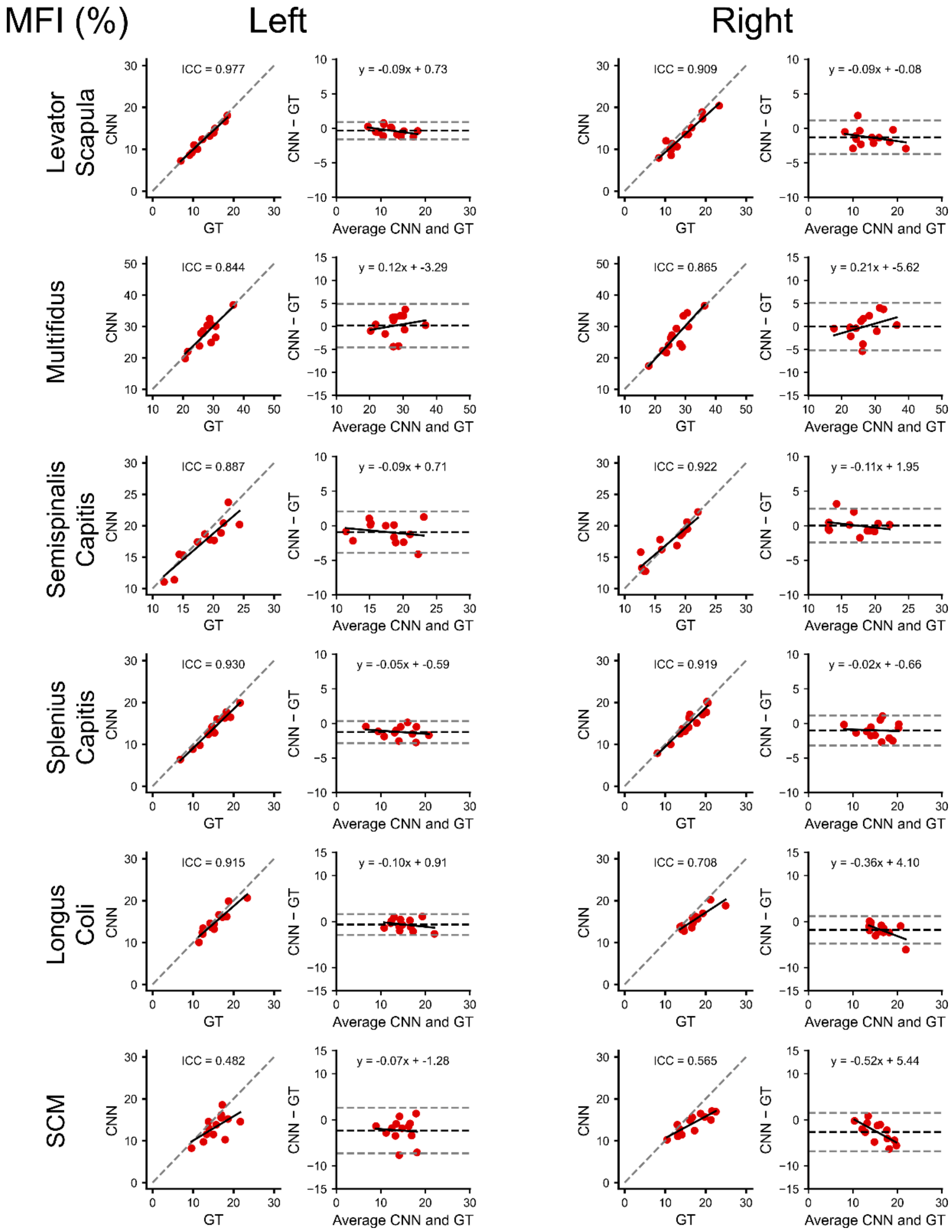

Abbreviation: ICC = intraclass correlation coefficient ($ICC_{2,1}$). In the correlation plot, the dashed gray line represents perfect correlation, and the solid black line represents the best fit line. In the Bland-Altman plots, the dashed black and gray lines indicate the mean difference (i.e., bias) ± 1.96 × standard deviation (i.e., 95% limits of agreement).

Supplementary Figure S4. Interrater reliability and accuracy for muscle volume (mm^3) between the convolutional neural network (CNN) model and the ground truth (GT) in the testing dataset (n = 13), assessed by correlation and Bland-Altman plots for each muscle or muscle group.

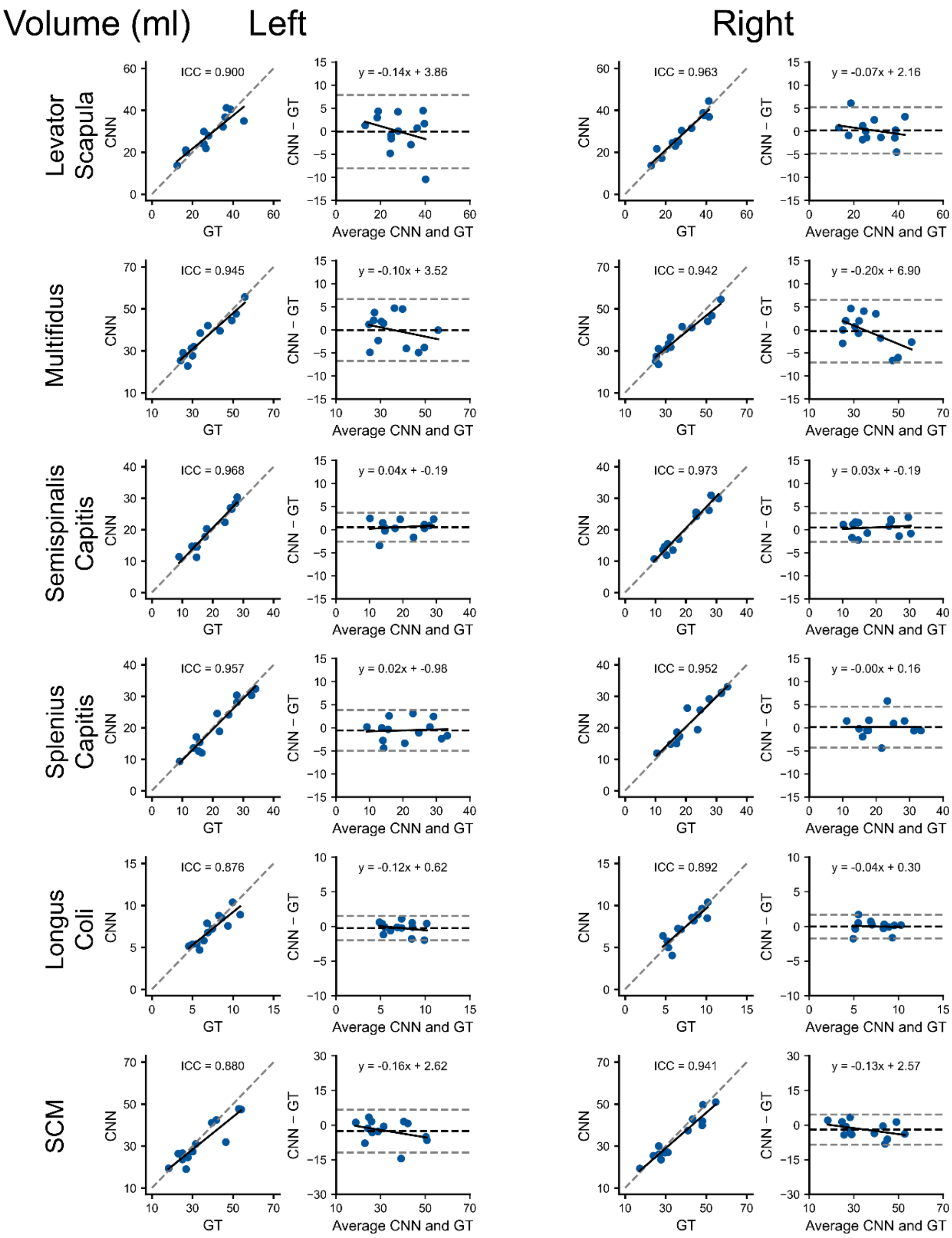

Abbreviation: ICC = intraclass correlation coefficient ($ICC_{2,1}$). In the correlation plot, the dashed gray line represents perfect correlation, and the solid black line represents the best fit line. In the Bland-Altman plots, the dashed black and gray lines indicate the mean difference (i.e., bias) ± 1.96 × standard deviation (i.e., 95% limits of agreement).
